# Peiminine Protects Dopaminergic Neurons from Inflammation-Induced Cell Death by Inhibiting the ERK1/2 and NF-κB Signalling Pathways

**DOI:** 10.3390/ijms19030821

**Published:** 2018-03-12

**Authors:** Guangxin Chen, Juxiong Liu, Liqiang Jiang, Xin Ran, Dewei He, Yuhang Li, Bingxu Huang, Wei Wang, Dianfeng Liu, Shoupeng Fu

**Affiliations:** College of Animal Science and Veterinary Medicine, Jilin University, Changchun 130062, China; cgxkc@outlook.com (G.C.); juxiong@jlu.edu.cn (J.L.); 17843105106@163.com (L.J.); ranxin21001x@163.com (X.R.); m13144303829@163.com (D.H.); yhli9915@mails.jlu.edu.cn (Y.L.); huangbingxu16@mails.jlu.edu.cn (B.H.); wangwei@jlu.edu.cn (W.W.); liudf@jlu.edu.cn (D.L.)

**Keywords:** peiminine, Parkinson’s disease, microglia, ERK1/2, AKT, NF-κB

## Abstract

Neuroinflammation, characterized marked by microglial activation, plays a very important role in the pathogenesis of Parkinson’s disease (PD). Upon activation, pro-inflammatory mediators are produced by microglia, triggering excessive inflammatory responses and ultimately damaging dopaminergic neurons. Therefore, the identification of agents that inhibit neuroinflammation may be an effective approach for developing novel treatments for PD. In this study, we sought to investigate whether peiminine protects dopaminergic neurons by inhibiting neuroinflammation. We evaluated the effects of peiminine on behavioural dysfunction, microglial activation and the loss of dopaminergic neurons in a rat model of lipopolysaccharide (LPS)-induced PD. BV-2 cells were pretreated with peiminine for 1 h and then stimulated with LPS for different times. Then, inflammatory responses and the related signalling pathways were analysed. Peiminine markedly attenuated behavioural dysfunction and inhibited the loss of dopaminergic neurons and microglial activation in the LPS-induced PD rat model. In BV-2 cells, peiminine significantly decreased LPS-induced expression of the pro-inflammatory mediators TNF-α, IL-6 and IL-1β, COX-2 and iNOS by inhibiting the phosphorylation of ERK1/2, AKT and NF-κB p65. Based on these results demonstrated that peiminine has a role in protecting dopaminergic neurons in the LPS-induced PD rat model by inhibiting neuroinflammation.

## 1. Introduction

Parkinson’s disease (PD) is one of the most common neurodegenerative diseases, second only to Alzheimer’s disease (AD) [1]. PD is characterized by the loss of dopaminergic neurons in the substantia nigra pars compacta (SNpc) and the formation of α-synuclein-containing protein aggregates, termed Lewy bodies, in the surviving neurons [2,3]. Although age and neurotoxins are established risk factors, and smoking has been suggested to be a protective factor, the definite aetiology of PD remains unknown [4]. However, neuroinflammation has recently been identified as playing a very important role in the pathogenesis of PD [5,6]. Although normal neuroinflammation is important for protecting the central nervous system (CNS), uncontrolled and prolonged neuroinflammation is potentially harmful and can cause cellular damage [7]. Microglia are the resident macrophages in the CNS [8] responsible for immune surveillance and responding to diseases and injuries in the CNS. Although immune cells can cross through the blood brain barrier (BBB) in patients with neurodegenerative diseases [9,10], the BBB limits the entry of cell into the CNS under normal physiological conditions [11]. Thus, microglia act as the main effector cells during the progression of neuroinflammation. Therefore, the identification of agents that inhibit microglial activation may be an effective approach for developing new treatments for PD.

Peiminine is an alkaloid extracted from *Fritillaria ussuriensis* and has been widely used in herbal remedies in China, Turkey, Pakistan, Japan and other southeast Asian countries [12,13,14]. *F. ussuriensis* and its extracts have been used to mitigate pulmonary fibrosis [15], tuberculosis [16], acute lung injury [17] and lung cancer [18]. Peiminine suppresses the production of pro-inflammatory cytokines in phorbol 12-myristate 13-acetate (PMA) and calcium ionophore-stimulated HMC-1 cells and LPS-treated RAW 264.7 cells [19,20]. However, reports on the anti-inflammatory effects of peiminine on the CNS have not been published. Therefore, the aim of this study was to evaluate the anti-inflammatory activity of peiminine in an LPS-induced PD rat model in vivo and the anti-inflammatory mechanisms of peiminine in microglia in vitro. Peiminine promoted anti-inflammatory responses in the LPS-induced PD rat model by inhibiting the ERK1/2, AKT and NF-κB signalling pathways. These experiments expand our understanding of the biological function of peiminine and implicate this alkaloid as a strong therapeutic candidate to prevent or slow the progression of PD.

## 2. Results

### 2.1. Peiminine Improves LPS-Induced Behavioural Dysfunction

**Figure 1:**
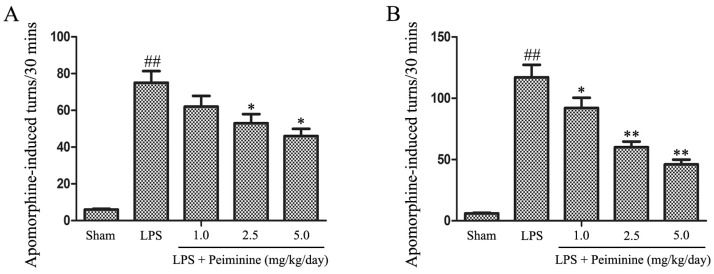
Peiminine improves LPS-induced motor dysfunction. Rats were pretreated with peiminine (1.0, 2.5 or 5.0 mg/kg/day) for three days prior to the intranigral injection of LPS for 28 days. The number of turns induced by apomorphine after two weeks (**A**) and four weeks (**B**) in the LPS-induced PD rat model is shown (*n* = 8 rats per group). ^##^ *p* < 0.01 compared to the sham group, * p < 0.05 and ** p < 0.01 compared to the LPS groups.

We initially investigated the effects of peiminine on behavioural dysfunction in the LPS-induced PD rat model. LPS was injected into the right SNpc, which induces microglial hyper-activation and dopaminergic neurons damage. Unilateral injury of dopaminergic neurons causes a compensatory hyper-activation of dopamine receptors, leading to differences in receptor activity between the two sides of the brain; agonist-induced receptor activation results in rotational behaviour towards the injected side. Amphetamine, an indirect dopamine receptor agonist, causes rotational behaviour in the LPS-induced PD rat model [21]. The rotational behaviour assay was used to evaluate the extent of injury or recovery in the PD rat model. The peiminine treatment dramatically decreased amphetamine-induced rotational behaviour in the LPS-induced PD rat model (Figure 1).

### 2.2. Peiminine Inhibits the LPS-Induced Loss of Dopaminergic Neurons in the SNpc

**Figure 2:**
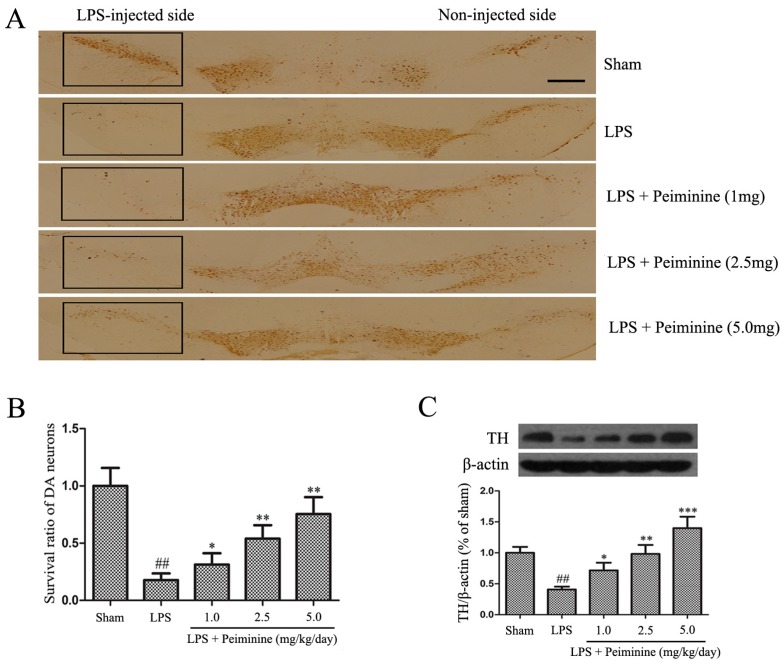
Peiminine inhibits the LPS-induced loss of dopaminergic neurons in the SNpc. PD model rats were decapitated on day 28 to obtain the midbrain. (**A**) Immunohistochemical staining for TH-positive cells; the magnification of the image is 4×, the scale bar represents 100 μm (*n* = 4 rats per group); (**B**) Ratio of surviving dopaminergic neurons (*n* = 4 rats per group); (**C**) TH expression (*n* = 4 rats per group). ^##^ *p* < 0.01 compared to the sham group, * *p* < 0.05, ** *p* < 0.01 and *** *p* < 0.001 compared to the LPS group.

Tyrosine hydroxylase (TH) is a rate-limiting enzyme that is broadly expressed in dopaminergic neurons for the synthesis of catecholamines, which are required for dopamine production [22]. Consequently, the amount of TH reflects the extent of dopaminergic neuronal loss in the SNpc. Immunohistochemical staining revealed a dramatic decrease in the number of TH-positive cells in the LPS group compared with that in the sham group, and peiminine attenuated the LPS-induced loss of TH-positive cells in a dose-dependent manner (Figure 2A,B). To further investigate the protective effects of peiminine on dopaminergic neurons, we examined levels of the TH protein in the SNpc. The Western blotting results indicated that peiminine significantly increased the LPS-induced decease in the level of the TH protein in the SNpc of model rats in a dose-dependent manner (Figure 2C). Based on these data, peiminine protected dopaminergic neurons in the LPS-induced PD rat model.

### 2.3. Peiminine Suppresses LPS-Induced Microglia Activation

**Figure 3:**
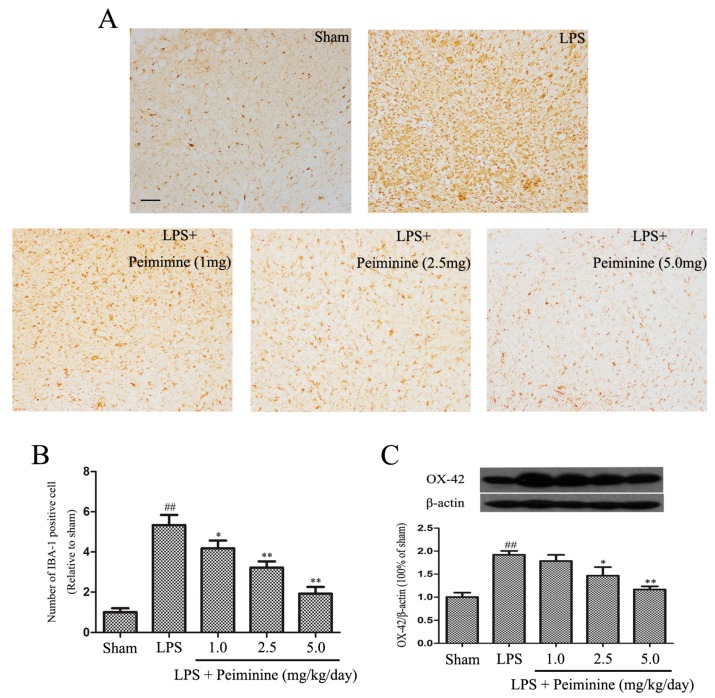
Peiminine suppresses the LPS-induced activation of microglia. PD model rats were decapitated on day 28 to obtain the midbrain. (**A**) The morphological changes of the microglia in the SN as shown by IBA-1 immunostaining. Representative photomicrographs of the SN area are shown. The magnification of the images is 40× and the scale bar represents 400 μm (*n* = 4 rats per group); (**B**) Number of IBA-1-positive cells (*n* = 4 rats per group); (**C**) OX-42 expression (*n* = 4 rats per group). ^##^ *p* < 0.01 compared to the sham group, * *p* < 0.05 and ** *p* < 0.01 compared to the LPS group.

Microglial activation is associated with the loss of dopaminergic neurons in the LPS-induced PD rat model [23]. IBA-1 is a marker of microglial activation and has been used to analyse the degree of microglial activation [21]. Hence, the number of IBA-1-positive cells reflects the percentage of activated microglia. Compared with the sham group, LPS significantly increased the number of IBA-1-positive cells in the SNpc, and the remaining ramified microglia were transformed into an amoeboid morphology. Peiminine dramatically decreased the number of IBA-1-positive cells in a dose-dependent manner (Figure 3A,B). Next, we measured the expression of another well-known marker of activated microglia, CD11b (OX-42) [24]. The Western blot results showed marked increase in OX-42 levels following treatment with LPS, and the peiminine treatment significantly decreased OX-42 expression in a dose-dependent manner (Figure 3C).

### 2.4. Peiminine Inhibits the LPS-Induced Inflammatory Response in BV-2 Cells

**Figure 4:**
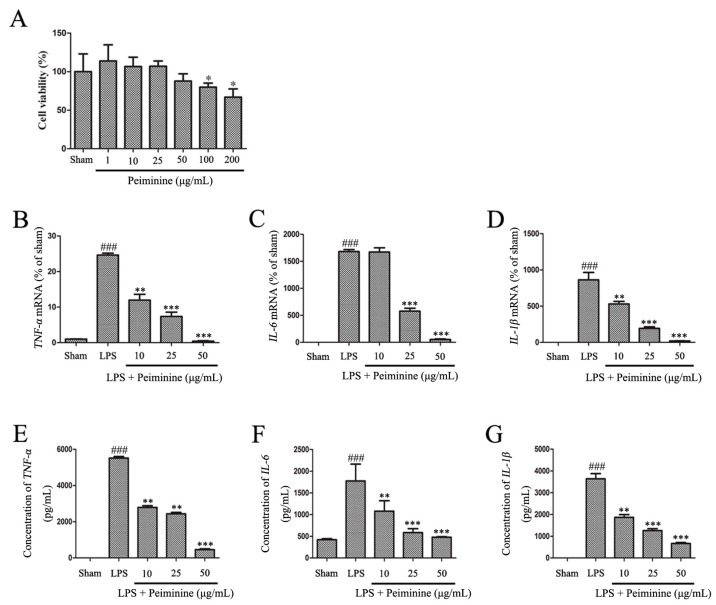
Peiminine inhibits the LPS-induced inflammatory response in BV-2 cells. (**A**) The effects of peiminine (1, 10, 25, 50, 100 and 200 μg/mL) on the viability of BV-2 cells. BV-2 cells were pretreated with various concentrations of peiminine (10, 25 or 50 μg/mL) for 1 h, then stimulated with LPS for 4 h. The expression of the *TNF-α* (**B**), *IL-6* (**C**) and *IL-1β* (**D**) mRNA was determined using quantitative real-time PCR (*n* = 3). Cells were pretreated with various concentrations of peiminine (10, 25 or 50 μg/mL) for 1 h, stimulated with LPS for 12 h, and the levels of the TNF-α (**E**), IL-6 (**F**) and IL-1β (**G**) proteins in media supernatants were measured using ELISA (*n* = 3). ^###^ *p* < 0.001 compared to the sham group, * *p* < 0.05 compared to the sham group, ** *p* < 0.01 and *** *p* < 0.001 compared to the sham group.

**Figure 5:**
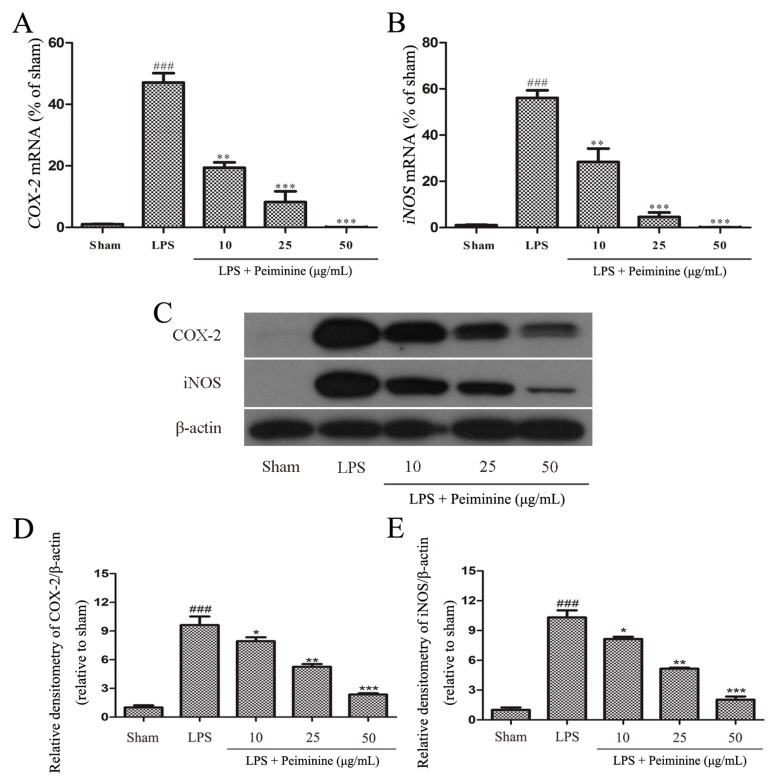
Peiminine inhibits the LPS-induced inflammatory response in BV-2 cells. BV-2 cells were pretreated with peiminine (10, 25 or 50 μg/mL) for 1 h and then stimulated with LPS for 4 h. (**A**,**B**) The expression of the *iNOS* and *COX-2* mRNAs was examined by quantitative real-time PCR (*n* = 3); (**C**–**E**) levels of the iNOS and COX-2 proteins were measured by Western blotting (*n* = 3). ^###^ *p* < 0.001 compared to the sham group, * *p* < 0.05, ** *p* < 0.01 and *** *p* < 0.001 compared to the LPS group.

Based on the results from the experiments described above, peiminine protected dopaminergic neurons in the LPS-induced PD rat model by inhibiting microglial activation. We examined the effects of peiminine on LPS-induced BV-2 cells to investigate the anti-inflammatory mechanism of peiminine. First, we evaluated the effects of peiminine on the viability of BV-2 cells. Peiminine had no effects on BV-2 cell viability at doses of 1, 10, 25 or 50 μg/mL, but significantly decreased cell viability at doses of 100 and 200 μg/mL. Hence, 50 μg/mL peiminine was the highest concentration that did not cause cell death (Figure 4A). LPS was applied to BV-2 cells as an in vitro inflammation model, and peiminine was added at various concentrations to determine whether peiminine exerted anti-inflammatory effects on BV-2 cells. LPS significantly increased the expression of the genes encoding the pro-inflammatory cytokines *TNF-α*, *IL-6* and *IL-1β*, and peiminine dramatically decreased the expression of these cytokine genes in a dose-dependent manner (Figure 4B–D). We further examined the effects of peiminine on the levels of the TNF-α, IL-6 and IL-1β proteins in media supernatants and found that peiminine treatment significantly decreased the LPS-induced secretion of TNF-α, IL-6 and IL-1β (Figure 4E–G). The pro-inflammatory cytokines TNF-α, IL-1β and IL-6 and the pro-inflammatory enzymes iNOS and COX-2 play very important roles in the process of inflammation [25,26]. Therefore, we measured the effects of peiminine on the mRNA and protein levels of pro-inflammatory enzymes in LPS-treated BV-2 cells using quantitative real-time PCR and Western blotting. Peiminine significantly decreased the mRNA (Figure 5A,B) and protein levels of pro-inflammatory enzymes (Figure 5C–E) in a dose-dependent manner.

### 2.5. Peiminine Suppresses LPS-Induced NF-κB p65 Phosphorylation in BV-2 Cells

**Figure 6:**
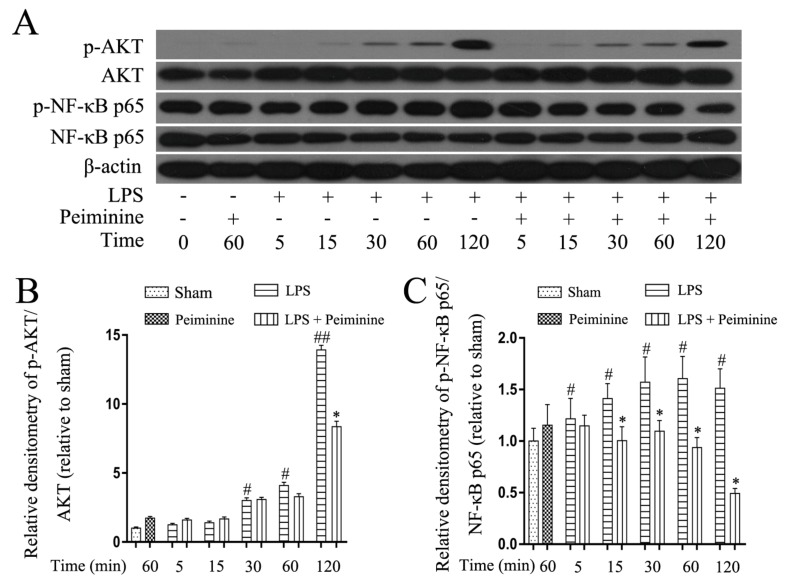
Peiminine suppresses the LPS-induced phosphorylation of NF-κB p65 in BV-2 cells. Cells were pretreated with peiminine (50 μg/mL) for 1 h and then stimulated with LPS (1 μg/mL) for 5, 15, 30, 60 or 120 min. Western blot results (**A**) (*n* = 3). Peiminine inhibited the LPS-induced phosphorylation of NF-κB p65 and AKT (**B**,**C**). ^#^ *p* < 0.05 and ^##^ *p* < 0.01 compared to the sham group, * *p* < 0.05 compared to the LPS group at the same time point.

NF-κB is activated by Toll-like receptors and plays a pivotal role in regulating the expression of pro-inflammatory mediators. The early development of neurodegenerative diseases is often accompanied by microglia activation. The NF-κB signalling pathway was analysed in LPS-treated BV-2 cells to explore the mechanism by which peiminine modulates microglial activation. Cells were pre-treated with or without peiminine for 1 h and then treated with LPS for 5, 15, 30, 60 or 120 min. Peiminine significantly suppressed LPS-induced phosphorylation of NF-κB p65 and AKT (Figure 6A–C), a kinase upstream of NF-κB.

### 2.6. Peiminine Suppresses LPS-Induced ERK1/2 Phosphorylation in BV-2 Cells

**Figure 7:**
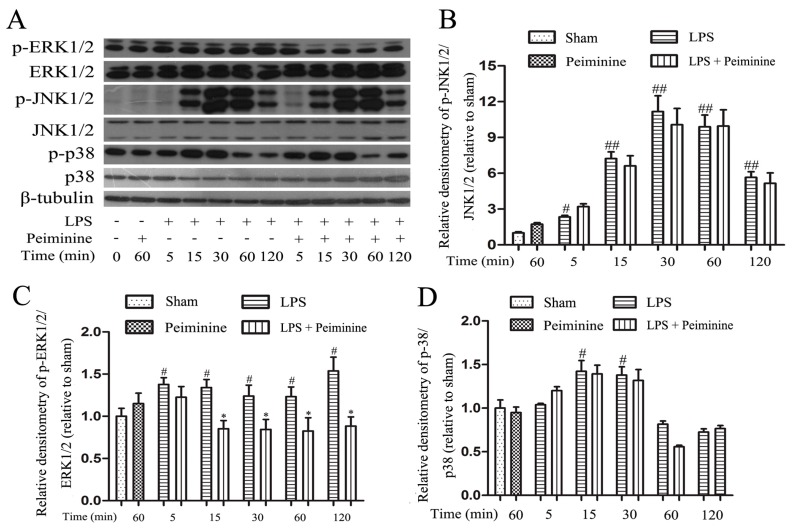
Peiminine suppresses LPS-induced ERK1/2 phosphorylation in BV-2 cells. Cells were pretreated with peiminine (50 μg/mL) for 1 h and then stimulated with LPS (1 μg/mL) for 5, 15, 30, 60 or 120 min. Western blot results (**A**) (*n* = 3). Peiminine inhibited LPS-induced ERK1/2 phosphorylation (**C**), but failed to inhibit the phosphorylation of JNK1/2 and p38 (**B**,**D**). ^#^ *p* < 0.05 and ^##^ *p* < 0.01 compared to the sham group, * *p* < 0.05 compared to the LPS group at the same time point.

Mitogen-activated protein kinase (MAPK) signalling pathways are known to play a very important role in the regulating of inflammatory responses [27]. Therefore, we examined the effects of peiminine on the phosphorylation of ERK1/2, JNK1/2 and p38 by Western blotting to elucidate the mechanism by which peiminine modulates the activation of BV-2 cells. LPS significantly increased the phosphorylation of ERK1/2, JNK1/2 and p38 (Figure 7A). Pretreatment with peiminine significantly inhibited LPS-induced ERK1/2 phosphorylation (Figure 7C), but failed to inhibit the phosphorylation of JNK1/2 and p38 (Figure 7B,D).

## 3. Discussion

Innate immunity plays a significant role in the pathogenesis of neurological disorders [28]. Although this view was initially met with scepticism, aberrant inflammatory responses in the CNS are now accepted to contribute to neuronal dysfunction [29]. Inflammation promotes debris clearance and tissue repair, and thus, this process is considered a beneficial physiological response of the CNS. However, sustained inflammatory signalling interferes with other functions of the CNS [6] and is important for the development of neuroinflammation [30]. This finding is consistent with reports that hyper-activated microglia secrete NO, PGE2, TNF-α, IL-1β and IL-6, which contribute to the inflammatory response in patients with neurodegenerative diseases [31,32]. Hence, the inhibition of microglial hyper-activation may be a potential therapeutic strategy for PD.

Peiminine attenuated bleomycin-induced lung injury in rats in a previous study [15]. Peiminine also significantly inhibits phorbol 12-myristate 13-acetate (PMA) and calcium ionophore-stimulated inflammation in HMC-1 cells [19]. Imperialine and verticinone significantly suppress the expression of pro-inflammatory mediators in LPS-treated RAW 264.7 macrophages [20]. Furthermore, other flavonoids, such as licochalcone A, quercetin and galangin, have been implicated as anti-neuroinflammatory agents in subjects with degenerative diseases [33,34,35]. Thus, the flavonoid peiminine may have a potential role in inhibiting neuroinflammation and display the potential to alleviate the progression of PD. The LPS-induced PD rat model has not only been widely used to study the role of neuroinflammation in dopaminergic neurons degeneration but has also been used to screen anti-neuroinflammatory drugs [10]. Therefore, in the present study, we investigated the anti-inflammatory effects of peiminine on the LPS-induced PD rat model.

The prominent motor symptoms in PD, which include rigidity, tremors, and bradykinesia, are due to the degeneration of dopaminergic neurons in the SN [36]. Amphetamine, an indirect dopamine receptor agonist, causes rotational behaviour in the LPS-induced PD rat model [21]. In this study, peiminine markedly decreased the amphetamine-induced rotational behaviour, the results indicated that peiminine improve the LPS-induced dopaminergic neurons loss. TH is a rate-limiting enzyme that is broadly expressed in dopaminergic neurons in the CNS [22] and synthesizes catecholamines, which are required for dopamine production [37]. Thus, the TH content has widely been used to assess the degree of damage to the dopaminergic system. Immunohistochemical staining revealed a dramatic decrease in the number of TH-positive neurons in the LPS group compared to the sham group, and peiminine inhibited this loss in a dose-dependent manner. We examined the levels of the TH protein in the SNpc by Western blotting to further investigate the effects of peiminine on TH and found that peiminine significantly increased TH expression in the SNpc of the PD rat model. Based on these data, peiminine inhibited the LPS-induced loss of dopaminergic neurons in the PD rat model. Microglial activation is associated with the loss of dopaminergic neurons in the LPS-induced PD model [23]. Consequently, we examined the expression of IBA-1 and OX-42, biomarkers of microglial activation [21]. LPS treatment dramatically increased the expression of IBA-1 and OX-42, whereas peiminine inhibited the upregulated expression of these proteins in a dose-dependent manner, suggesting that peiminine suppressed LPS-induced microglial activation. Thus, peiminine protected dopaminergic neurons in the LPS-induced PD rat model by inhibiting microglial activation.

BV-2 cells are the most commonly used alternative model to primary microglia [38]. Therefore, we used BV-2 cells to investigate the anti-neuroinflammatory mechanism of peiminine. First, we investigated the effects of peiminine on the production of pro-inflammatory cytokines in LPS-treated BV-2 cells and found that peiminine dramatically inhibited the expression of the TNF-α, IL-6 and IL-1β mRNAs. We also examined the levels of the TNF-α, IL-6 and IL-1β proteins in culture supernatants and found that peiminine significantly decreased the secretion of TNF-α, IL-6 and IL-1β. Based on these results, peiminine suppressed LPS-induced production of pro-inflammatory cytokines, consistent with the findings reported in previous studies [19,20]. Next, we explored the effects of peiminine on LPS-induced production of pro-inflammatory enzymes (COX-2 and iNOS) in BV-2 cells. Peiminine significantly decreased LPS-induce COX-2 and iNOS production. Therefore, peiminine suppressed LPS-induced inflammatory responses in BV-2 cells. The expression of inflammatory mediators in activated microglia is regulated by several intra-glial signalling pathways, including the MAPK and NF-κB signalling pathways [39]. Flavonoids have been reported to exert neuroprotective effects via their interactions with critical neuronal/glial intracellular signalling pathways controlling the levels of inflammatory mediators [40]. For example, quercetin and licochalcone A exert neuroprotective effects by regulating the phosphorylation of components of the MAPK and NF-κB signalling pathways [34,35]. Similarly, the flavonoid peiminine inhibits LPS- and PMAC-induced inflammation by reducing the activation of NF-κB and MAPK signalling pathways [15,19,20]. In the present study, peiminine significantly inhibited the phosphorylation of ERK1/2, AKT and NF-κB p65, but had no effect on the phosphorylation of JNK1/2 and p38, indicating that peiminine inhibits the inflammatory response by inhibiting the phosphorylation of ERK1/2, AKT and NF-κB p65.

## 4. Materials and Methods

### 4.1. Animals and Treatments

Two-month-old female Wistar rats weighting approximately 250 g were provided by the Center of Experimental Animals of the Baiqiuen Medical College of Jilin University (Changchun, China) and maintained under specific pathogen-free conditions. All the rats were provided with food and water ad libitum and housed on a 12-h light-dark cycle. Rats were randomly divided into five groups of 8 rats per group as follows: the sham group; the LPS-treated group, in which rats were anaesthetized with sodium pentobarbital (45 mg/kg) and then the right SNpc was injected with 10 μg of LPS at a concentration of 5 μg/μL; and the peiminine groups, in which peiminine was dissolved in normal saline and injected intraperitoneally at a dosage of 1, 2.5, or 5 mg/kg once per day beginning 3 days prior to LPS injection, for a total of 28 days. The studies were performed in accordance with the guidelines established by the Jilin University Institutional Animal Care and Use Committee (approved on 27 February 2015, Protocol No. 2015047).

### 4.2. Rotational Behaviour Assay

After the LPS injection, apomorphine was intraperitoneally injected into the rats to assess lesion severity, and the rotational behaviour assay was performed in the second and fourth weeks, as previously described [41,42]. Briefly, the rats were allowed to acclimate to a circular test arena for a short time before they were injected with the dopamine receptor agonist apomorphine. Beginning 5 min after apomorphine administration, the number of turns was recorded over a 30-min testing period.

### 4.3. Cells and Treatments

BV-2 cells were cultured in complete Dulbecco’s Modified Eagle’s Medium (DMEM) (Gibco, Grand Island, NY, USA) supplemented with 10% (*v*/*v*) foetal bovine serum (FBS) (Clark, Claymont, DE, USA) and incubated at 37 °C in a humidified atmosphere of 5% CO_2_. Cells were starved by incubating them with serum-free DMEM for 4 h to reduce mitogenic effects. Cells were pretreated with various concentrations of peiminine (mol. wt. 429.64, HPLC ≥ 98.0%) (Shanghai Yuanye Bio-Technology Co., Ltd., Shanghai, China) for 1 h and then stimulated with LPS (Sigma-Aldrich, St. Louis, MO, USA) for various time periods.

### 4.4. Immunohistological Analysis

Midbrains were washed with phosphate-buffered saline (PBS), fixed with 4% formaldehyde, embedded in paraffin and sectioned (3 μm). Immunohistological staining was performed using the following primary antibodies: anti-tyrosine hydroxylase (TH) (1:1000; Abcam, Cambridge, CA, USA) and anti-ionized calcium-binding adaptor molecule-1 (IBA-1) (1:200, Proteintech, Chicago, IL, USA). The numbers of TH- and IBA-1-positive cells in the SNpc were counted by three researchers, and the averages are reported.

### 4.5. Cell Viability Assay

The MTT assay was used to determine the effects of peiminine on BV-2 cells viability. Briefly, BV-2 cells were seeded in 96-well plates at a density of 1 × 10^4^ cells/well and incubated overnight at 37 °C in a 5% CO_2_ atmosphere. Various concentrations of peiminine were added to the cells and incubated for 24 h. MTT (5 mg/mL) was then added to each well and incubated at 37 °C for 4 h. DMSO (100 μL) was added to each well to dissolve the crystals, and absorbance was measured at 570 nm. Five replicates were conducted for each concentration of peiminine.

### 4.6. Quantitative Real-Time PCR

Total RNA was extracted from BV-2 cells using TRIzol (Invitrogen, Carlsbad, CA, USA) according to the supplier’s protocol, and the cDNA templates were generated using a commercial RT-PCR kit (Takara Shuzo Co., Ltd., Kyoto, Japan). The cDNA templates were amplified with the SYBR Green QuantiTect RT-PCR Kit (Roche, South San Francisco, CA, USA) using specific primers to evaluate the mRNA levels of various genes, and each samples was analysed in triplicate. The sequence of primers used in this investigation are shown in Table 1.

### 4.7. ELISA

BV-2 cells were seeded in 24-well plates and cultured overnight. The cells were starved in serum-free medium for 4 h to reduce mitogenic effects. The cells were pretreated with 10, 25 or 50 μg/mL peiminine for 1 h and then stimulated with LPS (1 μg/mL) for an additional 12 h. Subsequently, the medium was collected and centrifuged at 12,000× *g* for 10 min. Levels of the TNF-α, IL-6 and IL-1β proteins were measured using a commercial ELISA kits obtained from BioLegend (San Diego, CA, USA).

### 4.8. Western Blot Analysis

Cells were treated with LPS (1 μg/mL) after pretreatment with 50 μg/mL peiminine for 1 h. Cells were harvested after 5, 15, 30, 60 or 120 min in cold PBS and homogenized in lysis buffer (Beyotime Inst. Biotech, Beijing, China). After centrifugation at 12,000× *g* at 4 °C, the protein concentrations in the supernatants were quantified with a bicinchoninic acid protein assay kit (Beyotime Inst. Biotech). For each sample, 50 μg of protein were resolved by 12% SDS-polyacrylamide gel electrophoresis (SDS-PAGE) and transferred onto immunoblot polyvinylidene difluoride membranes (Chemicon International, Millipore, Billerica, MA, USA). After transfer, the membranes were blocked with 5% non-fat milk in Tris-buffered saline supplemented with 0.1% Tween-20 (TBS-T, pH 7.4) at room temperature for 2 h. Subsequently, the membranes were incubated overnight at 4 °C with primary antibodies against OX-42 (1:1000), TH (1:1000), COX-2 (1:1000), iNOS (1:2000) (Abcam, Cambridge, CA, USA), phospho-NF-κB p65 (1:1000), NF-κB p65 (1:1000), phospho-AKT (1:2000), AKT (1:2000), phospho-p38 (1:2000), p38 (1:1000), phospho-ERK1/2 (1:2000), ERK1/2 (1:2000), phospho-JNK1/2 (1:1000), JNK1/2 (1:2000), (Cell Signaling Technology, Danvers, MA, USA), and β-actin (1:2000) (Santa Cruz Biotechnology Inc., Santa Cruz, CA, USA). The membranes were washed 4 times (15 min each) with TBS-T and incubated with a horseradish peroxidase-labelled secondary goat anti-rabbit (1:2000) or goat anti-mouse antibody (1:2000) (Santa Cruz Biotechnology Inc., Santa Cruz, CA, USA) at room temperature for 1 h. Next, membranes were washed 4 times with TBS-T and visualized with an enhanced chemiluminescence ECL kit (Applygen Inst. Biotech, Beijing, China).

### 4.9. Statistics

The results are expressed as the means ± SD. Data were measured with Shapiro-Wilk-test then, differences between groups were compared using one-way analysis of variance (ANOVA) followed by the least significant difference test. The statistical software package SPSS 12.0 (SPSS Inc., Chicago, IL, USA).

## Figures and Tables

**Table 1:** The primer sequences of TNF-α, IL-1β, IL-6, iNOS, COX-2 and β-actin.

Gene	Sequence		Length (bp)
*TNF-α*	F: 5′-CCACGCTCTTCTGTCTACTG-3′	R: 5′-GCTACGGGCTTGTCACTC-3′	145
*IL-1β*	F: 5′-TGTGATGTTCCCATTAGAC-3′	R: 5′-AATACCACTTGTTGGCTTA-3′	131
*IL-6*	F: 5′-AGCCACTGCCTTCCCTAC-3′	R: 5′-TTGCCATTGCACAACTCTT-3′	156
*iNOS*	F: 5′-CACCCAGAAGAGTTACAGC-3′	R: 5′-GGAGGGAAGGGAGAATAG-3′	186
*COX-2*	F: 5′-AGAGTCAGTTAGTGGGTAGT-3′	R: 5′-CTTGTAGTAGGCTTAAACATAG-3′	170
*β-actin*	F: 5′-GTCAGGTCATCACTATCGGCAAT-3′	R: 5′-AGAGGTCTTTACGGATGTCAACGT-3′	147

## Abbreviations

PD	Parkinson’s disease
SNpc	Substantia nigra pars compacta
TH	Tyrosine hydroxylase
IBA-1	Ionized calcium-binding adaptor molecule-1
